# Decoding the Endocrine Code of Skeletal Muscle: Myokines, Exerkines, and Inter-Organ Crosstalk in Metabolic Health and Disease

**DOI:** 10.3390/cells15040318

**Published:** 2026-02-08

**Authors:** Young-Sool Hah, Jeongyun Hwang, Seung-Jun Lee, Seung-Jin Kwag

**Affiliations:** 1 Department of Surgery, Institute of Medical Science, Gyeongsang National University College of Medicine, Jinju 52727, Republic of Korea; yshah@gnu.ac.kr; 2 Biomedical Research Institute, Gyeongsang National University Hospital, Jinju 52727, Republic of Korea; 3 Department of Convergence Medical Sciences, Gyeongsang National University, Jinju 52725, Republic of Korea; dkdl10252@gnu.ac.kr (J.H.); 0789zxc@gnu.ac.kr (S.-J.L.)

**Keywords:** myokines, exerkines, skeletal muscle, endocrine organ, exercise, insulin resistance, extracellular vesicles, FGF21, GDF15, myostatin

## Abstract

Skeletal muscle is increasingly recognized as a dynamic endocrine and paracrine organ that communicates with distal tissues through a diverse secretome of peptides, proteins, metabolites, and extracellular vesicles (EVs), collectively referred to as myokines and exerkines. Beyond cataloging individual factors, emerging evidence suggests that muscle-derived signals can convey information through an integrated, context-dependent “endocrine code”—a pattern defined by secretion kinetics, co-released signal combinations, delivery modalities, and target-tissue receptor landscapes. This review synthesizes current evidence on (i) conceptual and experimental criteria for defining bona fide myokines, (ii) mechanisms governing myokine expression, processing, and release across exercise modes and physiological states, and (iii) major muscle–organ axes that connect physical activity to systemic metabolic homeostasis, immune remodeling, tissue regeneration, and neurocognitive adaptation. We further discuss non-protein mediators such as lactate, succinate, and β-aminoisobutyric acid, and highlight EVs as a multiplexed delivery modality whose interpretation requires stringent isolation, contamination controls, and functional validation. Finally, we evaluate translational opportunities—including biomarker panels, therapeutic targeting of the myostatin/activin, fibroblast growth factor 21 (FGF21), and growth differentiation factor 15 (GDF15) pathways, and precision exercise prescriptions informed by multi-omics and artificial intelligence—while emphasizing analytical standardization, causal validation, and transparent reporting as prerequisites for clinical impact.

## 1. Introduction

Skeletal muscle, constituting approximately 40% of adult body mass, has evolved from being viewed merely as a mechanical apparatus for locomotion to being recognized as a bona fide endocrine organ. Over the past two decades, convergent physiological and omics data have established that muscle communicates with distal tissues through a diverse secretome, collectively termed myokines. Foundational work identified muscle contraction as the primary stimulus for this endocrine function [1,2], with Interleukin-6 (IL-6) emerging as the archetype linking exercise to systemic metabolic regulation [3,4]. Subsequent research has expanded this concept from single protein mediators to a coordinated ‘myokinome’—and more broadly, ‘exerkines’ encompassing non-protein signals—that varies dynamically with exercise modality, nutritional state, and disease context [5,6,7,8].

The endocrine capacity of skeletal muscle is rooted in its immense structural scale and vascular plasticity, enabling the state-dependent exchange of signals with the circulation. While classic syntheses viewed these pathways as secondary to contractile biology [9,10], it is now clear that muscle-derived signals orchestrate a multi-axis communication network. These signals act locally to regulate myogenesis and angiogenesis, while simultaneously exerting endocrine effects on adipose tissue, the liver, the pancreas, the brain, and the immune system. This systemic integration underlies the concept of exercise as a ‘polypill’ capable of conferring broad cardiometabolic protection [11].

Despite expanding catalogs of putative myokines, establishing causality remains a critical challenge. Many candidates are not exclusively muscle-derived, and their transport in extracellular vesicles (EVs) complicates the attribution of tissue provenance [12,13]. Furthermore, interpretation is often confounded by the dependence of these signals on sampling timing and assay methodology [14,15]. These complexities necessitate a conceptual shift from static cataloging to frameworks that explicitly account for source attribution, exposure kinetics, and target engagement.

In this review, we propose the term “endocrine code” as a testable framework to describe the syntax of muscle-to-organ signaling. Moving beyond simple abundance, we propose that the biological impact of muscle-derived factors is determined by an integrated pattern defined by secretion kinetics, combinatorial release, delivery modalities, and the receptor landscape of target tissues.

Encoding is multidimensional, governed by (i) kinetics (pulsatile vs. sustained exposure); (ii) combinatorial structure (co-released modules rather than isolated factors); (iii) delivery modality (soluble proteoforms vs. EV-encapsulated cargo); and (iv) context gating by factors such as nutritional status and training history [12,13,14,15,16]. Conversely, decoding is dictated by the availability of receptors and the state of the downstream pathway in the target tissue. Together, these elements determine whether a signal is interpreted as adaptive (e.g., an acute exercise pulse) or maladaptive (e.g., chronic elevation leading to ‘noise’) [13,17,18,19,20].

**Figure 1:**
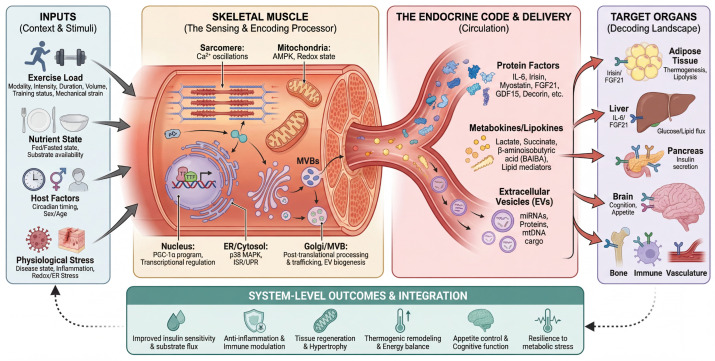
The “endocrine code” of skeletal muscle: inputs (exercise and context) are transduced by intracellular sensing and regulatory layers into coordinated outputs (protein myokines, metabokines/lipokines, and extracellular vesicle cargo) that act on target organs to shape system-level phenotypes.

This framework yields testable predictions for experimental design. It suggests that phenotypes attributed to single factors often reflect coordinated, partially redundant ensembles, motivating network-level and multi-analyte analyses rather than single-marker narratives [7,8,20]. Crucially, it frames “myokine resistance” not merely as a defect but as a breakdown in encoding/decoding fidelity, in which chronically elevated baseline signals reduce the signal-to-noise ratio and promote receptor/pathway desensitization and impaired downstream responsiveness [9,20]. This concept is consistent with well-described context dependence in myokine biology—for example, IL-6 can exert adaptive effects in acute exercise settings, whereas prolonged exposure can contribute to impaired insulin signaling and cellular insulin resistance in relevant target tissues [21,22,23]. Building on this perspective, we synthesize mechanistic evidence for the generation and decoding of these signals, identifying methodological bottlenecks and outlining translational opportunities in biomarker development and precision exercise medicine. Figure 1 provides a schematic overview of this encoding and decoding logic across muscle–organ axes. Specifically, the figure illustrates how physiological inputs (e.g., exercise modality/intensity/duration, nutritional state, aging) are sensed by skeletal muscle and transduced into multi-layer “encoded” outputs (proteins/peptides, metabolites, extracellular vesicles) with distinct amplitudes and temporal dynamics. These circulating signals are then “decoded” by recipient tissues according to receptor expression and intracellular context, generating organ-specific phenotypic outputs (metabolic, immune, regenerative, and neurocognitive adaptations).

## 2. Defining the Myokinome and Establishing Causality

The term ‘myokine’ is often used broadly to describe any factor whose concentration changes with exercise. For clarity, we distinguish three related concepts: (i) Myokines are broadly defined as cytokines, peptides, or growth factors synthesized and expressed by muscle fibers that exert autocrine, paracrine, or endocrine effects. This definition has evolved to include not only canonical cytokines (e.g., IL-6) but also novel peptides encoded by open reading frames within long non-coding RNAs, thereby representing a specific subset of the muscle secretome regulated by contraction [1,2]. (ii) Exerkines represent a comprehensive category of signaling moieties released into circulation in response to acute or chronic exercise. Unlike myokines, exerkines are not restricted to skeletal muscle origin but include factors released by the liver (hepatokines), adipose tissue (adipokines), neurons (neurokines), and extracellular vesicles (EVs). As highlighted by recent reviews on osteoarthritis and systemic crosstalk, this term encompasses proteins, nucleic acids (miRNA, mtDNA), lipids, and metabolites that collectively mediate the systemic adaptation to physical activity [5,6,7,8,24]. (iii) Adipo-myokines are factors produced by both muscle and adipose tissue that participate in bidirectional crosstalk [1,8,12]. Additional exercise-responsive candidates reported in the literature include chemokines/cytokines (e.g., IL-8/CXCL8, CXCL1), growth and remodeling factors (e.g., IL-15), and other peptide mediators, underscoring that Table 1, Table 2, Table 3 and Table 4 are representative rather than exhaustive. This distinction is important because tissue provenance, delivery mode, and receptor context can differ substantially, even when the same molecule is detected in circulation.

From an experimental perspective, a rigorous myokine designation requires more than a correlation between exercise and plasma abundance. Mechanistic studies have employed combinations of muscle contraction models (e.g., electrically stimulated myotubes) and arteriovenous sampling across active muscle beds to link contraction to secretion [103,104]. Complementary secretome and genetic perturbation approaches can then be used to test whether candidate factors are necessary and/or sufficient for a downstream phenotype [105,106].

Human cell secretome experiments further indicate that insulin-resistant myotubes release a qualitatively different mixture of bioactive factors that can influence pancreatic β-cell function, underscoring the importance of disease context and target-tissue state [107].

In practice, rigorous source attribution is often the rate-limiting step. Many proteins detected in plasma after exercise are abundant intracellular constituents or extracellular matrix fragments that may rise due to membrane disruption, proteolysis, or altered clearance rather than regulated secretion. Accordingly, candidate identification should be coupled with secretion-competent models (e.g., primary myotubes, electrically stimulated contraction systems) and complemented by evidence of physiologically relevant exposure and target engagement [104,105]. Human studies can strengthen provenance by combining time-resolved sampling with arteriovenous balance across active limbs, alongside transcriptomic/proteomic atlases that support tissue enrichment. These approaches, when integrated with functional perturbations in model systems, help prevent circular inference in which any exercise-responsive factor is assumed to be a myokine solely based on correlation [106,107].

To guide future research, we propose a set of operational criteria for defining bona fide myokines (Box 1).

Box 1 **Operational criteria for bona fide myokines.**

 A factor can be considered a bona fide myokine when at least four of the following six criteria are met, with Criteria #1 and #2 serving as essential prerequisites to establish muscle provenance:

(1) Demonstrable expression in skeletal muscle fibers or myotubes, with contraction- and/or metabolic stress-responsive regulation.
(2) Evidence of secretion from muscle (e.g., increased release in conditioned media, EV preparations, or arteriovenous gradients).
(3) Identifiable receptor(s) and signaling pathway(s) in target tissue(s) consistent with physiological concentrations.
(4) Causal linkage to phenotype by muscle-specific genetic manipulation, neutralization, or receptor perturbation.
(5) Consideration of confounding sources (adipose, immune, liver) and pre-analytical/analytical variability (sampling time, processing, assay specificity).
(6) For EV-associated factors, adherence to minimal reporting and characterization standards (e.g., particle metrics, marker panels, and functional controls) [14,15,19].

Finally, high-throughput proteomics has uncovered hundreds of contraction-regulated secreted proteins, but annotating these as functional myokines requires orthogonal validation and careful control of cell death, serum contamination, and batch effects. Systematic secretome analyses across model systems suggest that only a subset of detected proteins exhibits robust contraction regulation, and even fewer demonstrate endocrine actions in vivo [34,104,105]. Accordingly, interpretative frameworks that emphasize networks and signaling modules rather than single mediators may better capture the biology of muscle–organ communication [20]. Given the vast number of potential myokines identified in proteomic screens, this review does not aim to be an exhaustive catalog. Instead, we focus on select mediators that best illustrate the “endocrine code” principles: defined secretion kinetics, established distal receptors, and proven contribution to inter-organ crosstalk.

## 3. Regulation of Myokine Expression and Secretion

Myokine production is tightly coupled to the physiological ‘state’ of the muscle fiber, integrating mechanical load, energetic stress, calcium flux, redox state, and endocrine inputs. Across individuals, the magnitude and direction of myokine responses are further shaped by training status, sex, age, baseline inflammation, and the sampling window relative to the exercise bout [14,15,35]. These considerations help explain why some myokines (e.g., IL-6) show robust and reproducible responses, whereas others (e.g., irisin) remain controversial due to assay sensitivity, protein processing, and timing effects [4,15,108].

### 3.1. Exercise Modality and Dose as Primary Determinants

Distinct exercise modalities ‘encode’ specific physiological priorities into the circulation. Aerobic and endurance exercise typically elicits significant increases in myokines associated with energy sensing and substrate mobilization. IL-6 levels rise in proportion to the duration and intensity of exercise and are potentiated by low muscle glycogen, positioning it as a metabolic sensor that coordinates glucose and lipid flux during prolonged exercise [25,26,27]. Endurance training can also remodel the basal myokine milieu, with studies identifying apelin as a contraction-regulated factor that plays a role in oxidative adaptation [59].

Resistance and hypertrophy-focused training, in contrast, tend to emphasize local remodeling signals that support myogenesis, extracellular matrix turnover, and tissue growth. Key examples include the regulation of decorin, which antagonizes myostatin signaling to facilitate hypertrophy [38,109], and leukemia inhibitory factor (LIF), a contraction-induced factor that stimulates myocyte proliferation [39,110]. Additionally, Interleukin-15 (IL-15) has been identified as an anabolic myokine that lowers visceral fat while promoting muscle protein accretion. Mechanistically, IL-15 has been reported to bias myofiber protein balance toward anabolism by engaging Akt-mTOR signaling and limiting FOXO-driven ubiquitin-proteasome programs, thereby supporting hypertrophy while attenuating atrophy-associated transcriptional signatures [53]. IL-15 may also act through muscle-adipose crosstalk to improve substrate partitioning, indirectly reinforcing anabolic remodeling during resistance training [53]. Musclin (Osteocrin), an activity-responsive peptide, supports endurance capacity and may facilitate homology-directed repair in muscle, though its primary role is linked to endurance adaptation [53]. These signals largely act in an autocrine/paracrine manner but can spill over into circulation to reflect the tissue’s remodeling state.

High-intensity interval training (HIIT) produces a hybrid signature, engaging both ‘endurance-like’ metabolic pathways (AMPK/p38 MAPK axes) and robust ‘stress-response’ programs (e.g., *PGC-1α* induction) due to the marked energetic perturbations it induces. While training mode clearly influences myokine expression patterns, heterogeneity in protocols remains a barrier to meta-analytic synthesis [34,35].

To facilitate translational interpretation, Table 1 maps these modality-dependent signatures to their corresponding dominant signaling outcomes.

### 3.2. Intracellular Transcriptional and Post-Transcriptional Gateways

At the intracellular level, PGC-1α acts as a central transcriptional coactivator integrating calcium-dependent signaling, β-adrenergic inputs, and energy sensing to drive oxidative remodeling and fiber-type specification [111,112,113]. Seminal work identified a PGC-1α-dependent myokine program that promotes thermogenic remodeling of white adipose tissue, illustrating how transcriptional states in muscle can propagate endocrine outcomes [29].

More recently, mitochondrial integrated stress responses have been linked to myokine induction, including FGF21 and GDF15, which serve as circulating markers of mitochondrial perturbation [70,114,115]. Beyond transcription, secretion can be regulated by proteolytic processing, membrane trafficking, and EV biogenesis. Muscle-derived EVs contain proteins, lipids, mRNAs, and microRNAs capable of remodeling recipient cells, and they may provide a protected vehicle for labile signals that would otherwise be degraded in plasma [13,116,117]. These post-transcriptional mechanisms reinforce why transcript abundance alone is often an unreliable proxy for endocrine exposure, necessitating coordinated multi-omics approaches to fully decode the muscle secretome [70,114].

## 4. Autocrine and Paracrine Functions Within Skeletal Muscle

### 4.1. Control of Muscle Mass, Regeneration, and Remodeling

Within a muscle, a balance between anabolic and catabolic programs governs adaptation to training, injury, and aging. Myostatin (a TGF-β family member) is a key negative regulator of muscle mass, and its expression is detectable in both normal and diseased human skeletal muscle [55,56]. Therapeutic and experimental efforts targeting the myostatin/activin axis have provided proof of concept for increasing lean mass, but translation to functional outcomes has been variable across indications, emphasizing that mass gain alone may not equate to improved performance [109].

Myokines that modulate the myostatin pathway illustrate the complexity of local signaling. Decorin is regulated by contraction and has been linked to muscle hypertrophy, potentially through interactions with myostatin signaling [38]. Meanwhile, adaptations to loading also reflect global proteostasis pathways such as the ubiquitin–proteasome system, which shapes the turnover of sarcomeric and regulatory proteins in both health and disease [118].

Muscle regeneration additionally depends on crosstalk among myofibers, satellite cells, immune populations, and the extracellular matrix. Emerging literature suggests that EVs participate in this regenerative ‘orchestra’, transferring instructive cues that can prime myoblast differentiation and coordinate repair processes [119,120].

### 4.2. Local Metabolic Reprogramming and Insulin Sensitivity

Contraction rapidly alters muscle metabolism via calcium signaling, energetic stress, and mechanical cues, leading to AMPK activation and downstream remodeling. AMPK is a key node linking energetic stress to improved mitochondrial function and insulin sensitivity, and its activation is a canonical feature of endurance and interval training adaptation [121]. At the receptor/signaling interface, muscle-specific regulation of insulin receptor abundance and downstream signaling can be influenced by nuclear receptor pathways such as PPARβ/δ, highlighting an additional layer of metabolic control [122].

Some myokines also exert autocrine effects that feed back on muscle substrate handling. IL-6 can activate AMPK in skeletal muscle and regulate fat oxidation, but chronic elevations are associated with insulin resistance and inflammatory states, underscoring the importance of temporal dynamics and context [27,94,123]. These observations align with broader frameworks explaining insulin resistance as an emergent phenotype of inflammation, lipid overload, and impaired signaling, rather than a single-factor defect [123].

## 5. Systemic Myokine-Mediated Crosstalk Across Organs

Myokines mediate systemic effects through endocrine actions on distal tissues, frequently converging on energy balance, inflammation resolution, and tissue-specific remodeling. Importantly, the “endocrine code” framework suggests that many phenotypes attributed to exercise reflect the coordinated action of multiple myokines and exerkines acting in concert, rather than a single dominant effector [7,8]. Below, we synthesize key inter-organ axes, emphasizing how these signals are integrated (Table 2).

### 5.1. Muscle–Adipose Axis: Adipose Browning, Lipolysis, and Inflammation

The muscle–adipose axis is central to systemic insulin sensitivity and thermogenic capacity. The identification of irisin (a cleavage product of *FNDC5*) as a PGC-1α-dependent mediator that promotes brown-fat-like programs in white adipocytes catalyzed extensive investigation into myokine-driven browning [29]. Subsequent human studies have reported associations between irisin and adipocyte differentiation and metabolic phenotypes; however, heterogeneity in assays and population characteristics has sustained debate about the effect size and physiological relevance [108,124]. However, recent syntheses reaffirm irisin’s role not only in adipose tissue but also in protecting distal organs such as the kidneys and lungs against inflammatory and fibrotic stress, broadening its scope beyond thermogenesis [52].

In addition to irisin, the small metabolite β-aminoisobutyric acid (BAIBA) can induce adipose browning and influence hepatic lipid metabolism, linking muscle metabolism to systemic energy balance [63]. Meteorin-like (Metrnl) has also been implicated as a contraction-responsive hormone that regulates immune-cell homeostasis and insulin sensitivity, suggesting that muscle-to-adipose communication is intertwined with immune regulation [66]. Collectively, these factors illustrate how distinct molecular classes—proteins and metabolites—can converge on adipose tissue remodeling. Furthermore, myostatin, traditionally viewed as a local negative regulator of muscle mass, has been shown to inhibit browning and lipolysis in adipose tissue, effectively coordinating energy partitioning between muscle and fat stores [57].

### 5.2. Muscle–Liver Axis: Hepatic Glucose and Lipid Flux

The muscle–liver axis is traditionally viewed through the Cori cycle and substrate shuttling, but endocrine signaling introduces additional regulatory layers. IL-6 can promote hepatic glucose production during acute exercise while supporting lipid oxidation and glycemic control at the whole-body level, consistent with its dual endocrine and immune-modulatory roles [4,27]. Acute exercise can also induce FGF21 expression in mice and humans, aligning mitochondrial and hepatic lipid handling with energetic stress [69]. Given the therapeutic interest in FGF21 analogues for obesity-related diseases and non-alcoholic steatohepatitis (NASH), endocrine pharmacology has advanced rapidly [68,125]. However, disentangling the contributions of muscle versus liver to circulating FGF21 remains an active area of investigation and may influence the interpretation of trial readouts [126,127]. Similarly, while irisin was initially characterized for its browning effect, emerging evidence highlights its capacity to reduce hepatic lipogenesis and oxidative stress, reinforcing the hepatoprotective arm of the muscle–liver axis [52].

### 5.3. Muscle–Pancreas Axis: β-Cell Function and Insulin Secretion

Muscle secretomes can influence pancreatic islet biology. Conditioned media from insulin-resistant human myotubes has been shown to modulate pancreatic β-cell secretion, supporting the concept that ‘diabetogenic’ or ‘protective’ secretome states may participate in disease progression or remission [107]. These findings motivate a shift from single-factor biomarkers toward panels that capture secretome states and their downstream effects on insulin secretion and clearance.

### 5.4. Muscle–Brain Axis: Cognition, Mood, and Appetite Control

Exercise confers reproducible benefits on cognition and mood, and several muscle-derived factors have been implicated in mediating brain adaptation. Running-induced systemic cathepsin B secretion has been linked to memory function, and BDNF-dependent mechanisms connect physical activity to synaptic plasticity and cognition [31,32]. Animal studies further suggest that irisin can regulate cognitive function, potentially connecting muscle contraction to neurotrophic signaling [128].

Beyond cognition, muscle-derived signals may influence appetite and stress circuits. Circulating GDF15 has emerged as a marker of mitochondrial stress and is implicated in appetite regulation and cachexia-related phenotypes [70,114,115]. Specifically, GDF15 binds to the GFRAL receptor in the hindbrain to suppress food intake, while FGF21 modulates macronutrient preference and energy expenditure via central signaling, highlighting a conserved stress-response axis between muscle and the brain. An expanding literature connects exerkines, including lactate and other metabolites, to neurobiology and behavior, consistent with the view that metabolic intermediates can serve as signaling molecules (sometimes termed ‘lactormones’) [5,77,78].

### 5.5. Additional Axes: Bone, Immune System, and Cancer-Related Signaling

Myokine actions extend to bone and immune compartments, reinforcing the concept of whole-body remodeling. Exercise-induced anti-inflammatory effects are mediated by multiple pathways, including cytokine modulation, immune cell trafficking, and metabolic reprogramming of inflammatory states [94,129]. In parallel, myokines such as SPARC and decorin have been proposed to participate in exercise-associated colon cancer suppression and to regulate the tumor microenvironment, illustrating disease-relevant actions beyond classic metabolic endpoints [74].

## 6. Beyond Proteins: Metabokines, Lipokines, and Extracellular Vesicles

While protein myokines dominate the literature, a growing body of work supports a broader exerkine landscape that includes metabolites, lipids, and vesicular cargo. These mediators may be particularly important for rapid inter-organ signaling because they can change on minute-to-hour timescales and directly couple metabolic flux to endocrine information transfer.

### 6.1. Metabokines: Lactate, Succinate, and Related Intermediates

Lactate is a paradigmatic example of a metabolite that can function as a signaling molecule. Circulating lactate levels rise exponentially with high-intensity exercise (HIIT) and resistance training that exceeds the lactate threshold, serving not merely as a fuel but as a potent signaling molecule (“lactormone”). Mechanistic work suggests that lactate can stimulate IL-6 release from muscle during exercise, providing a link between glycolytic flux and cytokine signaling [49,77]. Recent conceptual syntheses propose ‘lactormone’ frameworks in which lactate coordinates multisystem adaptation, though causal in vivo evidence remains incomplete [78,79].

Conceptually, lactate illustrates how metabolic intermediates can transmit information not only as fuels but also as candidate ligands and chromatin-modifying signals. In addition to monocarboxylate transport, which redistributes lactate across tissues, lactate-responsive receptors and lactate-dependent regulatory marks have been proposed as mechanisms that could couple intensity-dependent glycolytic flux to downstream transcriptional programs [77,78]. In brain and immune compartments, lactate may function as both a substrate and a signal, complicating causal attribution unless tracer-based flux measurements and receptor perturbation are integrated into study designs [49,79].

Succinate has similarly emerged as a contraction-linked signal. Its release is triggered by hypoxic stress and high-energy turnover typical of high-intensity exercise. pH-gated succinate secretion from muscle has been proposed to regulate remodeling responses to exercise [37]. Succinate can signal through SUCNR1 (GPR91), and SUCNR1-dependent programs have been implicated in exercise-induced metabolic and immune adaptations across tissues [81,130,131]. Notably, circulating succinate appears to be responsive to exercise in humans; however, whether it primarily acts as a biomarker of mitochondrial flux or as a causal endocrine effector likely depends on concentration thresholds, receptor expression, and kinetics [132,133].

Succinate and related tricarboxylic acid (TCA) intermediates raise distinct interpretive issues because they can reflect both physiological flux and mitochondrial stress. Increases in circulating succinate may arise from muscle release, as well as from immune and adipose sources, and may be shaped by hypoxia- and inflammation-linked pathways [81,130]. Mechanistically, target-tissue SUCNR1 expression and local concentration thresholds likely determine whether succinate acts as an endocrine cue or a biomarker, underscoring the importance of arteriovenous sampling, receptor profiling, and dose–response analyses across various exercise modalities [131,132,133].

Beyond lactate and succinate, exercise-responsive metabolites such as β-aminoisobutyric acid (BAIBA) provide a complementary paradigm in which amino acid catabolic flux is linked to adipose and bone remodeling. Unlike lactate, BAIBA production is largely driven by PGC-1α-mediated *ADIB* expression, making it a signature of aerobic and endurance adaptation. Although untargeted metabolomics can nominate many candidates, only a subset has convergent evidence for regulated muscle production, release, and receptor- or pathway-mediated actions in vivo [63,66].

### 6.2. Lipokines and Lipid Mediators

Lipid-derived signals contribute to energy homeostasis and inflammatory tone and may interact with myokine programs. Exercise alters circulating lipids and lipid mediators, and lipokine biology intersects with muscle-derived signaling in regulating adipose tissue inflammation and insulin sensitivity [94,134]. Compared with protein myokines, lipid mediators face additional analytical challenges related to extraction, stability, and annotation, reinforcing the need for standardized workflows.

Exercise acutely perturbs lipid turnover and can generate lipid mediators that influence inflammation, insulin signaling, and vascular tone. Interpretation is challenging because many lipid classes undergo rapid post-sampling remodeling and because circulating levels integrate contributions from adipose tissue, liver, immune cells, and muscle [134].

Accordingly, robust lipokine biology benefits from standardized pre-analytical handling, harmonized lipidomics pipelines, and integration with isotope tracer strategies to distinguish endocrine signaling candidates from passive markers of substrate flux [94].

### 6.3. Extracellular Vesicles as Delivery Vehicles

**Figure 2:**
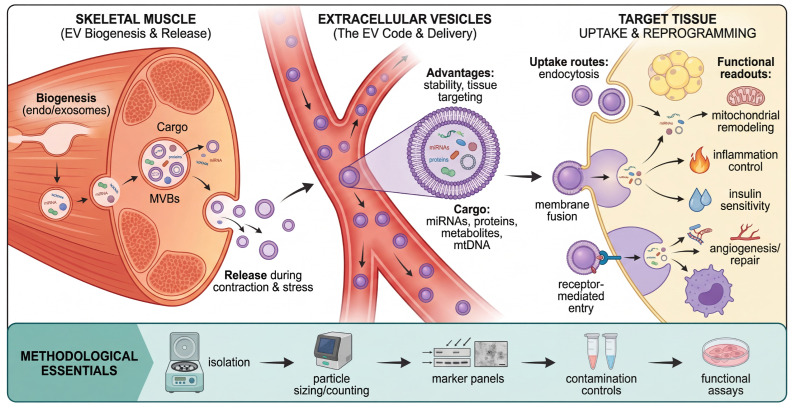
Extracellular vesicle (EV)-mediated muscle-to-organ communication. Skeletal muscle functions as a source of extracellular vesicles (EVs) that encapsulate a multiplexed ‘code’ of proteins, lipids, and nucleic acids (miRNAs, mtDNA). Unlike soluble myokines, this membrane-bound delivery system protects labile cargo from degradation and facilitates targeted uptake by distal organs such as adipose tissue and the liver. Upon internalization—via endocytosis or membrane fusion—EV cargo represses target genes (via miRNAs) or modulates metabolic flux, thereby transmitting complex adaptive signals. The lower panel emphasizes that establishing the functional relevance of this pathway requires adherence to rigorous methodological standards, including orthogonal isolation strategies and the exclusion of non-vesicular contaminants (for example, lipoproteins).

Extracellular vesicles (EVs) offer a complementary signaling modality to soluble myokines. Unlike free proteins, EVs encapsulate a multiplexed ‘code’ of proteins, lipids, and nucleic acids (miRNAs, mtDNA) within a lipid bilayer. This structure confers stability against degradation and facilitates targeted delivery to recipient cells [13,116,117]. To visualize this sophisticated signaling modality, Figure 2 outlines the journey of muscle-derived EVs—from their biogenesis in multivesicular bodies to their decoding by recipient tissues. This ‘packet-based’ communication enables the combinatorial transfer of metabolic and genetic information, providing a layer of complexity that extends beyond the capabilities of soluble factors.

Recent profiling studies suggest that EV cargo signatures shift with training status and disease, potentially modulating inflammatory tone, angiogenesis, and insulin sensitivity in distal organs [19,44,85]. However, the field faces significant technical hurdles, particularly the co-isolation of non-vesicular contaminants, such as lipoproteins and protein aggregates [86,120]. To progress from association to causality, it is imperative that studies adopt orthogonal isolation strategies and adhere to minimal reporting standards (MISEV). Future work must (i) include rigorous contamination controls, (ii) normalize EV doses in a biologically interpretable manner, and (iii) pair molecular profiling with functional assays to validate cargo transfer in vivo [85,120].

Beyond the well-characterized protein myokinome, the endocrine code is enriched by a diverse array of metabolites and vesicular signals. Table 3 provides a structured taxonomy of these non-protein mediators, contrasting the receptor-mediated actions of metabokines (such as lactate and succinate) [37,77,78,81] with the intracellular regulatory roles of EV-delivered miRNAs and mitochondrial DNA [19,85].

## 7. Myokine Network Disruption in Metabolic and Muscle-Related Pathologies

In pathological states such as obesity, type 2 diabetes (T2D), and sarcopenia, the precise “endocrine code” of skeletal muscle becomes dysregulated. This disruption occurs not only through altered secretion patterns (encoding errors) but also through impaired sensing by target tissues (decoding errors), collectively contributing to systemic metabolic inflexibility.

### 7.1. Obesity, Insulin Resistance, and Type 2 Diabetes

Obesity and type 2 diabetes (T2D) are characterized by chronic, low-grade inflammation, ectopic lipid accumulation, and altered endocrine environments that can alter the muscle secretome. Reviews of exercise-mediated myokine regulation in T2D emphasize that improvements in glycemic control likely arise from coordinated effects on muscle glucose uptake, hepatic glucose production, adipose inflammation, and pancreatic β-cell function [48,123]. Nutritional interventions (including caloric restriction and dietary composition) can further modulate the myokine milieu and may synergize with exercise, although separating the effects of weight loss from muscle-intrinsic signaling changes remains challenging [46].

Dietary energy restriction and weight-loss interventions can directly modulate circulating myokines, complicating cross-study comparisons in obesity when nutritional status is not harmonized. In adults with metabolic syndrome undergoing an 8-week hypocaloric intervention, circulating irisin levels decreased alongside improvements in glycemic indices, and higher baseline irisin levels predicted a larger metabolic response [135]. In a randomized controlled trial comparing three dietary patterns in metabolic syndrome, dietary composition was associated with distinct irisin trajectories, supporting diet quality as an independent source of myokine variability [136]. Weight-loss programs also report coordinated changes in serum myostatin and adiponectin that track remodeling of the skeletal muscle-to-visceral fat ratio, consistent with diet-responsive muscle–adipose crosstalk [137]. Cross-sectional data on obesity further indicate that serum myostatin is related to body-composition phenotypes in the context of concomitant adipokine signaling [138]. After Roux-en-Y gastric bypass surgery, baseline irisin levels were associated with subsequent weight loss, highlighting the potential prognostic utility of this biomarker but also underscoring the confounding impact of surgically induced energy deficit on biomarker interpretation [139].

### 7.2. Myokine Resistance and Exercise Non-Responsiveness

An emerging concept is ‘myokine resistance’, analogous to insulin or leptin resistance, where target tissues exhibit attenuated responses to otherwise beneficial signals. Potential mechanisms include receptor downregulation, altered receptor isoforms, chronic inflammatory interference with downstream signaling, and impaired EV uptake. Clinically, this may manifest as reduced metabolic benefit from standardized exercise programs in some individuals, motivating efforts to stratify responders and non-responders using biomarker and multi-omics approaches [17,18,20].

**Figure 3:**
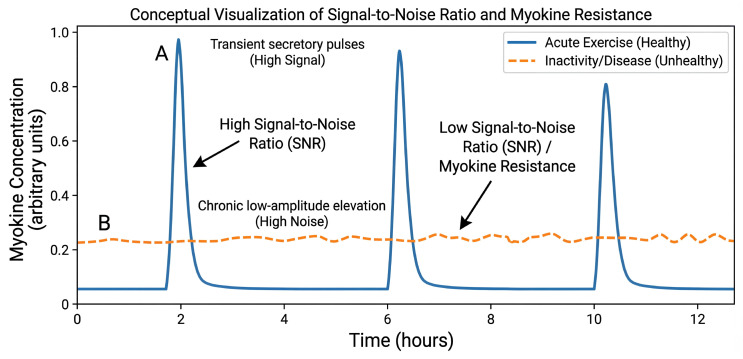
Temporal encoding of muscle-derived signals and the concept of myokine resistance. (A) In the healthy state (blue line), acute exercise acts as a high-fidelity encoder, generating transient, high-amplitude secretory pulses of myokines (for example, IL-6). This pulsatile pattern creates a high signal-to-noise ratio (SNR), allowing target tissue receptors to effectively sense the stimulus and undergo necessary resensitization during inter-pulse recovery periods. (B) In states of physical inactivity or chronic metabolic disease (orange dashed line), the secretory profile shifts toward a constitutive, low-amplitude elevation of stress signals. This chronic background “noise” significantly reduces the physiological SNR. Consequently, target tissues may exhibit maladaptive responses such as receptor downregulation and desensitization of downstream pathways (for example, impaired phosphorylation of signaling intermediates), leading to a failure to decode subsequent exercise stimuli—a phenomenon termed “myokine resistance.

From a coding perspective, resistance can be interpreted as a breakdown in the transfer of temporal and contextual information, where transient exercise-driven pulses are replaced by chronically elevated or noisy baseline signals. To conceptualize this failure, we propose a temporal encoding framework illustrated in Figure 3. In healthy physiology, skeletal muscle functions as a high-fidelity encoder, generating distinct secretory pulses that are readily detected by target tissues against a quiet background (Panel A in Figure 3). Conversely, in conditions of obesity or inactivity, the basal secretome is characterized by a chronic, low-grade elevation of inflammatory mediators. This persistent background ‘noise’ obscures the discrete signals of acute exercise, driving a maladaptive feed-forward loop of receptor desensitization and endocrine resistance (Panel B in Figure 3). Consequently, target tissues may downregulate receptors or rewire downstream pathways. This model predicts that restoring signal-to-noise (through exercise dose optimization, nutrition, circadian timing, or anti-inflammatory co-interventions) may be as important as increasing the absolute concentration of a single factor [17,20]. As an illustrative example, IL-6 is released from contracting human skeletal muscle during exercise; however, prolonged exposure to IL-6 can impair insulin signaling in hepatocyte models, consistent with the broader discussion of context-dependent myokine actions [4,22,140].

### 7.3. Sarcopenia, Cachexia, and Systemic Catabolism

In aging and chronic disease, loss of muscle mass and function (sarcopenia) can coexist with endocrine and inflammatory perturbations. Myokines have been implicated in muscle wasting states, and myostatin expression has been observed in cancer cachexia and related catabolic phenotypes [95,141]. Clinical biomarker studies suggest that circulating myostatin may reflect sarcopenic status in older individuals; however, interpretation requires attention to assay specificity and potential confounding comorbidities [97].

Systemic catabolism also interfaces with stress-associated myokines. GDF15 and FGF21 can increase in mitochondrial disease and other stress contexts, serving as both biomarkers and modulators of appetite and systemic energy balance [70,114,115]. Determining whether such factors are adaptive (promoting metabolic flexibility) or maladaptive (driving anorexia and tissue wasting) remains a key translational question that likely depends on exposure duration and tissue sensitivity [114,115]. Table 4 summarizes recurring patterns of myokine and exerkine dysregulation across common metabolic and muscle-related disease states, highlighting interpretive pitfalls and translational implications.

## 8. Translation: Biomarkers, Therapeutics, and Precision Exercise

### 8.1. Biomarker Panels and Analytical Standardization

Clinical biomarker interpretation requires explicit consideration of nutritional confounding and assay heterogeneity. In community-dwelling older adults, one cohort suggested that serum irisin has predictive value for sarcopenia [142], whereas another cohort reported that circulating irisin was largely independent of sarcopenia status and conventional muscle parameters [143]. For myostatin, sex-stratified analyses indicate that myostatin normalized to lean mass may associate with frailty and low appendicular muscle mass, with effect modification by sex and body composition [144].

The clinical deployment of myokines as biomarkers requires analytical rigor. Pre-analytical variability—including sampling time, proximity to exercise, anticoagulant choice, storage conditions, and freeze–thaw cycles—can introduce bias that exceeds many biological effect sizes [14]. Even for well-studied myokines, inter-assay variability and antibody specificity remain issues, motivating transparent reporting and, where feasible, the use of orthogonal quantification methods, such as mass spectrometry [15].

A practical strategy for translational studies is to employ multi-analyte panels that capture multiple axes (inflammation, mitochondrial stress, growth regulation, EV markers) rather than relying on single candidates. Such panels can be coupled to causal validation pipelines (muscle- and receptor-specific perturbations) and to longitudinal designs that distinguish acute exercise pulses from chronic baseline elevations [16,18,20].

Beyond assay performance, biomarker utility depends on harmonized sampling relative to exercise timing, feeding state, and circadian phase, because many candidates display rapid post-exercise kinetics and context-dependent baselines. Standardized protocols, transparent reporting, and pre-specified analytical plans are particularly important for multi-omic panels to prevent overfitting and to enable cross-cohort synthesis [14,16].

For practical implementation, Table 5 provides a methodological checklist that can be used to improve reproducibility, strengthen causal inference, and facilitate cross-study comparability in myokine and exerkine research.

### 8.2. Therapeutic Targeting of Myokine Pathways

Pharmacological modulation of myokine pathways is progressing along multiple fronts. Inhibition of the myostatin/activin axis has been pursued using antibodies and receptor decoys, including early agents evaluated in muscular dystrophy and other conditions [159]. Apitegromab (targeting pro/latent myostatin) and bimagrumab (ActRII antibody) represent later-generation approaches with clinical development programs in neuromuscular disease and metabolic indications, respectively [142,160,161]. Across programs, a recurring theme is that lean-mass increases do not always translate to proportional functional improvements, highlighting the need for endpoints that capture strength, quality of life, and metabolic health [109,161].

FGF21 has emerged as a highly active translational node at the intersection of mitochondrial stress, lipid handling, and systemic energy balance. Multiple engineered FGF21 analogues have advanced into clinical testing for obesity-related conditions [68,125]. Trials have reported improvements in lipid parameters and markers of steatohepatitis in selected populations [126,127]. However, dose translation across species and the relative contribution of muscle versus liver to circulating FGF21 remain important considerations when interpreting mechanisms and predicting on-target effects [69,162].

Emerging approaches include modulating stress-associated appetite pathways (e.g., GDF15 signaling) and developing EV-inspired delivery systems. For example, engineered nanobodies targeting GDF15 illustrate the broader interest in translating ‘exercise-like’ endocrine signals into therapeutics, although careful evaluation of benefit–risk trade-offs is necessary given the involvement of such pathways in anorexia and cachexia [114,163].

Translationally, pleiotropy presents both opportunities and risks: pathways such as myostatin/activin, FGF21, and GDF15 engage multiple tissues and can influence appetite, bone, and cardiovascular physiology. Accordingly, therapeutic development should prioritize tissue-selective mechanisms where possible, careful exposure–response modeling, and endpoints matched to the intended organ axis (e.g., insulin sensitivity, hepatic lipid flux, muscle strength) [68,109]. Table 6 provides a structured overview of translational strategies that target myokine and exerkine pathways, including key considerations for endpoint selection, tissue specificity, and safety monitoring.

### 8.3. Precision Exercise as a Systems Intervention

Exercise remains the most physiologically integrative approach to engaging the myokine network, but individual variability in response motivates a precision framework. Comparative studies and reviews suggest that aerobic, resistance, and interval training produce partially distinct myokine signatures, implying that the ‘endocrine code’ can be tuned through modality and dose [34,35]. In the near term, stratifying individuals based on baseline secretome states, inflammatory biomarkers, and metabolic phenotype may improve targeting of exercise prescriptions and combination therapies [46,48].

At the methodological frontier, integrating proteomics, transcriptomics, metabolomics, and EV profiling with AI-driven pattern recognition may enable predictive models of exercise responsiveness and disease risk. Recent work applying machine learning to muscle-derived molecular data illustrates the potential for decoding muscle–organ communication at scale, but reproducibility will depend on standardized pipelines and open data practices [17,18,165].

An actionable near-term goal is to define reproducible response signatures that stratify individuals into mechanistic “responder classes” (e.g., inflammatory-high versus mitochondrial-stress-high phenotypes) and then adapt exercise dose, modality, and timing accordingly. Such precision frameworks will likely require repeated-measures designs and iterative modeling rather than single time-point comparisons, and they must be benchmarked against clinically meaningful endpoints to avoid biomarker drift [16,18].

## 9. Conclusions and Research Priorities

The myokine field has progressed from identifying contraction-regulated candidates to appreciating skeletal muscle as a systems-level endocrine organ whose outputs encode information through molecular identity, kinetics, and delivery mode. To convert expanding catalogs into actionable biology, future work must prioritize causal validation, rigorous analytical standardization, and integrative models that capture inter-tissue feedback loops [16,18,20].

Three priorities are particularly pressing. First, the field needs agreed-upon experimental and reporting standards for EV-associated signaling and for high-throughput secretome studies, enabling cross-cohort comparability and meta-analytic synthesis [14,19]. Second, translational studies should adopt multimodal outcomes (metabolic, functional, and patient-reported) to reflect the network nature of the endocrine code [109]. These studies should also evaluate myokine-targeted therapies in combination with exercise and nutritional interventions, as supported by emerging clinical and mechanistic evidence [68,125]. Such combined approaches may be particularly valuable for metabolic liver disease and related conditions, where multiple FGF21 analogue programs are advancing [126,127]. Third, next-generation model systems—including organoids and organ-on-chip platforms—combined with spatial and single-cell omics may provide tractable settings for testing muscle–organ communication and myokine resistance mechanisms under controlled conditions [20,166].

Ultimately, the goal is not to reduce exercise biology to a single molecule, but to understand—and eventually manipulate—the myokine network to restore healthy inter-organ communication in metabolic disease, sarcopenia, and related conditions. A translationally mature ‘endocrine codebook’ will likely integrate classical myokines with metabokines, lipokines, and EV cargo while accounting for host factors such as the microbiome and circadian timing that modulate systemic responsiveness [167,168].

## Figures and Tables

**Table 1:** Exercise modality and dose shape distinct myokine/exerkine signatures and signaling outcomes.

Exercise Mode	Characteristic Signals (Myokines/Exerkines)	Dominant Signaling Outcome (The “Message”)	Interpretive Notes	References
Prolonged endurance (aerobic, >45 min)	Robust IL-6 increase; FGF21; Apelin; Lactate/FFA flux; Irisin (*FNDC5* cleavage); BDNF; Cathepsin B	Substrate Mobilization & Anti-inflammation (Coordinates fuel supply; acute immune modulation)	Contraction-driven secretion of IL-6, with endocrine-like systemic effects. Endurance exercise is also associated with circulating neurotrophic/protease signals linked to neurocognitive adaptation (e.g., BDNF/cathepsin B), potentially reflecting combined muscle and non-muscle contributions depending on context.	[25,26,27,28,29,30,31,32,33]
High-intensity interval training (HIIT)	Mixed cytokine/metabolite response; Lactate spikes; Succinate; Irisin; PGC-1α program	Rapid Metabolic Adaptation & Mitochondrial Stress (Signals for mitochondrial biogenesis and stress resilience)	Protocol heterogeneity is high; responses may resemble endurance ‘pulses’ with larger stress signatures. High metabolic flux drives accumulation of signaling metabolites (lactate, succinate).	[34,35,36,37]
Resistance/hypertrophy training	LIF, Decorin, IL-15; Suppression of Myostatin	Tissue Remodeling & Growth (Supports myogenesis, ECM turnover, and protein synthesis)	Local remodeling often dominates over large endocrine spikes; outcomes depend on volume and rest intervals	[38,39,40].
Concurrent (endurance + resistance)	Combined oxidative and remodeling signatures	Context-Dependent Interference or Synergy	Order and recovery can influence adaptation; endocrine outputs may be non-additive	[35,41,42]
Acute eccentric or damaging bouts	Inflammatory cytokines; Chemokines; EV release	Repair & Immune Recruitment (Initiates regeneration; transient pro-inflammatory phase)	May transiently elevate inflammatory markers; interpretation requires timing controls	[43,44,45]
Chronic training adaptations	Shift in basal secretome; Lower basal IL-6/TNF-α	Improved “Signal-to-Noise” Ratio (Lower basal inflammation preserves acute responsiveness)	Chronic adaptations may lower basal inflammation while preserving acute responsiveness	[46,47,48]

**Table 2:** Representative myokines and exerkines mediating inter-organ communication.

Signal (Class)	Primary Induction Context	Major Targets	Representative Actions	References
IL-6 (cytokine)	Prolonged/endurance exercise; low glycogen	Liver, adipose, immune cells	Coordinates substrate mobilization and contributes to anti-inflammatory reprogramming (acute); chronic elevation associates with insulin resistance	[4,22,49]
Irisin/*FNDC5*	PGC-1α program; endurance/HIIT	Adipose tissue; brain; liver; kidney; lung	Promotes thermogenic remodeling of white adipose tissue; implicated in neurotrophic adaptation (context-dependent); proposed roles in hepatic metabolic regulation and in kidney/lung protective pathways in exercise-related settings	[29,50,51,52]
Musclin (peptide)	Aerobic/Resistance exercise	Muscle; Heart; Adipose	Enhances physical endurance; protects against cardiac overload and fibrosis; promotes mitochondrial biogenesis.	[53,54]
Myostatin (TGF-β family)	Basal expression; catabolic states	Muscle (autocrine)	Negative regulator of muscle mass; systemically inhibits adipose tissue browning and lipolysis.	[55,56,57]
Decorin (ECM proteoglycan)	Resistance training; mechanical loading	Muscle (local); myostatin pathway	Modulates extracellular matrix; sequesters myostatin to enhance hypertrophy; potential onco-suppressive effects.	[38,58]
LIF (cytokine)	Contraction/loading	Muscle progenitors	Stimulates myocyte proliferation; supports regenerative adaptation	[39,40]
Apelin (peptide)	Contraction; endurance training	Muscle, vasculature	Supports oxidative adaptation and metabolic remodeling	[59,60,61]
BAIBA (metabolite)	Exercise-associated amino acid flux	Adipose, liver, bone	Induces browning and metabolic remodeling; emerging roles in bone cell survival	[62,63,64]
Metrnl (protein hormone)	Cold and exercise (context-dependent)	Immune cells, adipose	Regulates immune-cell homeostasis and promotes insulin sensitization	[65,66]
Cathepsin B (protease)	Running/endurance	Brain	Associated with exercise-induced memory benefits	[31,33]
BDNF (neurotrophin)	Exercise; muscle–brain signaling	Brain; potentially muscle	Supports synaptic plasticity and cognition; links physical activity to neurotrophic adaptation	[30,32]
FGF21 (mitokine/endocrine factor)	Energetic/mitochondrial stress; acute exercise	Liver, adipose, CNS	Acts centrally to regulate appetite; enhances insulin sensitivity in adipose tissue; modulates hepatic lipid flux.	[67,68,69]
GDF15 (stress cytokine)	Mitochondrial stress; disease states	Brainstem appetite circuits; systemic	Acts on brainstem (GFRAL) to suppress appetite; regulates lipolysis and systemic energy expenditure.	[70,71,72]
SPARC (matricellular protein)	Exercise responsiveness	Colon/epithelia; stromal cells	Proposed mediator of exercise-associated colon cancer suppression	[73,74]
Muscle-derived EVs (vesicular cargo)	Exercise; remodeling states	Multiple tissues	Multiplexed delivery of proteins/miRNAs/mtDNA; influences metabolism and regeneration	[13,75,76]

**Table 3:** The expanding exerkine landscape: non-protein mediators and EV cargo.

Mediator	Class/Delivery	Representative Targets	Notes	References
Lactate	Metabolite (‘lactormone’ candidate)	Muscle, liver, brain	Links glycolytic flux to cytokine release and systemic adaptation; may act as signal and substrate	[77,78,79,80]
Succinate	Metabolite; SUCNR1 ligand	Adipose, immune cells, muscle	pH-gated secretion and SUCNR1 signaling implicated in exercise adaptation; biomarker vs. effector context remains active debate	[37,81,82,83]
BAIBA	Metabolite	Adipose, liver, bone	Induces browning and metabolic remodeling; emerging bone-related actions	[62,63,64]
Musclin	Peptide (muscle-derived)	Cardiovascular system; metabolism	Reported to modulate physical endurance and cardiometabolic phenotypes	[15,84]
EV-associated miRNAs	Extracellular vesicle cargo	Multiple tissues	Candidate mediators of training adaptation; requires stringent EV isolation and functional validation	[19,85,86]
EV-associated proteins	Extracellular vesicle cargo	Adipose, liver, vasculature	Proteomic profiling suggests exercise-responsive EV signatures; target selection and uptake are key unknowns	[87,88,89]
EV-associated mtDNA	Extracellular vesicles/cell-free mtDNA	Immune system	Mitochondrial DNA can function as an inflammatory signal when released in vesicles or extracellular space	[44,90,91]
Exercise-derived exosomes (therapeutic concept)	EV-based intervention	Metabolic disease models	Endurance exercise-derived exosomes reported to treat metabolic disease in preclinical models; translation requires standardized manufacturing and safety evaluation	[43,76,92]

Note: This table distinguishes between ‘metabokines’ (metabolites acting as signaling ligands) and EV-associated cargo. While metabokines typically signal via cell-surface G-protein-coupled receptors (GPCRs), EV cargo requires cellular uptake to exert intracellular regulatory effects.

**Table 4:** Myokine network disruption across major pathophysiological contexts.

Condition	Typical Myokine/Exerkine Alterations	Implications for Phenotype	References
Obesity/T2D	Shift toward pro-inflammatory milieu; altered IL-6 signaling dynamics; variable irisin associations	Contributes to insulin resistance and impaired exercise responsiveness; motivates multi-marker panels	[46,48,51,93]
NAFLD/metabolic liver disease	Elevations in stress-associated factors (e.g., FGF21); altered lipid mediators	Potential biomarker utility and therapeutic targeting; muscle–liver source attribution remains important	[68,69,94]
Cancer cachexia	Myostatin and stress cytokines may rise; anorexia pathways engaged (e.g., TGF-β family, GDF15)	Muscle wasting and appetite dysregulation; requires careful benefit–risk evaluation for pathway modulation	[71,95,96]
Age-related sarcopenia	Changes in growth-regulatory signals (e.g., myostatin) and reduced anabolic responsiveness	Lean mass and function decline; clinical trials targeting activin/myostatin pathways show mixed functional outcomes	[97,98,99]
Inflammatory/autoimmune disease (example: multiple sclerosis)	Exercise-related endocrine and immune modulation intersects with neurological pathology	Supports personalized exercise as adjunct therapy; mechanistic work ongoing	[45,47,100]
Depression/Cognitive Decline	Blunted BDNF response; altered kynurenine pathway signaling	Impaired neuroplasticity and mood regulation; exercise may restore neurotrophic support	[30,32]
Chronic Kidney Disease (CKD)	Reduced circulating Irisin; elevated myostatin and inflammatory cytokines	Loss of renoprotection (fibrosis prevention); contributes to uremic sarcopenia and systemic inflammation	[24,52]
Chronic Obstructive Pulmonary Disease (COPD)	Dysregulated Irisin and oxidative stress markers; systemic inflammatory spillover	Disrupted muscle–lung axis; potential loss of anti-inflammatory buffering against pulmonary stress	[52]
Osteoarthritis and musculoskeletal comorbidity	Inflammation and altered muscle signaling may influence joint health and activity tolerance	Highlights need for integrated musculoskeletal–metabolic frameworks and tailored exercise prescriptions	[21,24,101,102]

**Table 5:** Methodological checklist for robust myokine/exerkine research and translation.

Methodological/Interpretive Issue	Best-Practice Recommendation	References
Context dependence and kinetics	Distinguish acute exercise pulses from chronic baseline elevations; predefine sampling windows aligned to mechanistic hypotheses	[145,146,147]
Causal attribution of ‘muscle-derived’ signals	Combine secretion evidence with muscle-specific perturbations and/or advanced human-relevant models (e.g., tissue-engineered muscle) to test necessity/sufficiency	[104,107,148]
Heterogeneity of exercise protocols	Report intensity, duration, modality, nutritional state, and training status; interpret findings within modality-specific signature literature	[34,147,149]
Pre-analytical and analytical variability	Standardize collection tubes, processing time, storage, and assay platforms; where possible, use orthogonal quantification	[14,15]
EV isolation and characterization	Follow minimal information standards; include controls for co-isolated proteins/lipoproteins and report particle metrics and marker panels	[19,85]
Biomarker translation and disease stratification	Validate candidate panels across cohorts and disease contexts; consider emerging myokine biomarkers (e.g., IL-7) and stress axis markers (FGF21/GDF15)	[150,151]
Synthesis of mechanistic and clinical literature	Use structured narrative frameworks and consolidate evidence on myokines in diabetes/insulin resistance and metabolic homeostasis	[106,152,153,154]
Field mapping and horizon scanning	Leverage bibliometric analyses and conceptual frameworks to identify emerging hotspots and gaps (e.g., ageing, EV cargo)	[155,156]
Controversies and assay debates (example: irisin)	Interpret associations with attention to assay specificity, population differences, and mechanistic plausibility; triangulate with multi-omics and receptor biology	[108,157]
Mechanistic breadth of IL-6 signaling	Account for IL-6 actions on lipolysis and insulin secretion/disposal across tissues when interpreting training studies	[22,28,158]

**Table 6:** Translational strategies targeting myokine/exerkine pathways.

Strategy/Agent	Target Pathway	Clinical/Preclinical Context	Key Considerations	References
Myo-029 (antibody)	Myostatin neutralization	Muscular dystrophy (early trials)	Lean-mass effects may not directly translate to functional gains; endpoint selection critical	[109,159]
ACE-031/ActRII decoys	Activin receptor ligand trap	Muscle wasting indications	Broad ligand binding may drive efficacy and off-target effects; safety monitoring essential	[118,159]
Apitegromab	Pro/latent myostatin	Spinal muscular atrophy and related neuromuscular disorders	Represents later-generation specificity; development pipeline evolving	[99,160]
Bimagrumab	ActRII antibody	Obesity/T2D, sarcopenia and myositis	Can increase lean mass and reduce fat mass; functional and metabolic endpoints vary by population	[142,161,164]
FGF21 analogs (e.g., pegozafermin and related)	FGF21 signaling	Metabolic liver disease/dyslipidemia	Promising metabolic effects; mechanism and source attribution require careful study; dose translation matters	[68,94,127]
GDF15 pathway modulation (e.g., engineered binders)	Stress-associated appetite regulation	Cachexia/anorexia biology; exploratory therapeutics	Potential to influence appetite and energy balance; benefit–risk depends on indication and dosing	[71,72,163]
EV-inspired or EV-based interventions	Multiplexed cargo delivery	Preclinical metabolic disease models	Manufacturing, characterization, biodistribution, and safety are major translational hurdles	[19,76,92]
Precision exercise prescriptions	Network-level intervention	Across cardiometabolic and musculoskeletal disease	Requires stratification and adaptive dosing; integration with nutrition and pharmacotherapy likely	[34,35,101]

## Data Availability

No new data were created or analyzed in this study. Data sharing is not applicable to this article.

## Abbreviations

The following abbreviations are used in this manuscript:

ActRII	Activin receptor type II
AMPK	AMP-activated protein kinase
BAIBA	β-aminoisobutyric acid
BDNF	Brain-derived neurotrophic factor
ECM	Extracellular matrix
EV	Extracellular vesicle
FGF21	Fibroblast growth factor 21
FFA	Free fatty acid
*FNDC5*	Fibronectin type III domain-containing protein 5
GDF15	Growth differentiation factor 15
GLP-1	Glucagon-like peptide-1
HIIT	High-intensity interval training
IGF-1	Insulin-like growth factor 1
IL-6	Interleukin-6
ISR	Integrated stress response
LIF	Leukemia inhibitory factor
MAPK	Mitogen-activated protein kinase
Metrnl	Meteorin-like
miRNA	microRNA
MISEV	Minimal Information for Studies of Extracellular Vesicles
mtDNA	Mitochondrial DNA
NASH	Non-alcoholic steatohepatitis
OSTN	Osteocrin (musclin)
PGC-1α	Peroxisome proliferator-activated receptor γ coactivator 1α
SEC	Size-exclusion chromatography
SOCS	Suppressor of cytokine signaling
SUCNR1	Succinate receptor 1 (GPR91)
T2D	Type 2 diabetes
TGF-β	Transforming growth factor-beta
